# Jehovah's Witness Needing Critical Care: A Narrative Review on the Expanding Arsenal

**DOI:** 10.1155/2024/1913237

**Published:** 2024-05-22

**Authors:** Ryan Davids, Gareth Robinson, Charmé Van Tonder, Jordan Robinson, Nadiyah Ahmed, Abdurragmaan Domingo

**Affiliations:** ^1^Department of Anaesthesiology and Critical Care, Stellenbosch University, Stellenbosch, Western Cape, South Africa; ^2^Department of Critical Care, University of Free State, Bloemfontein, South Africa; ^3^Department of Anaesthesiology and Perioperative Medicine, University of Cape Town, Cape Town, Western Cape, South Africa

## Abstract

Present day Jehovah's Witness (JW) religion accounts for 8.5 million followers. A tenant feature of the JW faith is religious objection to transfusions of blood and blood products. Interpatient variability, as it pertains to blood and blood products may occur; hence, a confidential interview will determine which products individual may consent to (Marsh and Bevan, 2002). This belief and practice place great restrictions on treating medical professionals in scenarios of life-threatening anaemia and active haemorrhage. The review to follow explores the physiological and pathophysiological consequences of severe anaemia. Non-blood transfusion practices are explored, many of which are potentially lifesaving. Particular attention is drawn to the evolving science involving artificial oxygen carriers and their use in emergency situations. A greater safety profile ensures its future use amongst religious objectors to be greatly beneficial. Intravenous iron supplementation has enjoyed a lively debate within the critical care community. A review of recent systematic and meta-analysis supports its use in the ICU; however, more investigation is needed into the complementary use of hepcidin.

## 1. Introduction

The Jehovah's Witness (JW) religion, founded by Charles Taze Russell in 1881, is a denomination of Christianity which holds to the core belief of non-trinitarianism and infallibility of the bible, with 8.5 million followers internationally [1].

Multiple biblical scriptures prohibit the “consumption” of blood, meaning patient refusal of blood products. This refusal of potentially lifesaving therapy has resulted in many conflicts between medical professionals and the JW followers.

This conflict is of particular importance in the critically ill where optimisation of blood flow and oxygen delivery is of paramount importance.

Intensive Care Units (ICU) are organised systems for the provision of care to critically ill patients suffering great physiological trespass and managed by specialised medical and nursing care. The aim of these units is to provide organ support. Blood and blood products are considered essential lifesaving therapies, particularly in anaemic patients requiring improvement of oxygen delivery.

The faith-based refusal of autologous or allogeneic blood transfusions, if blood is not in contact with the circulation, conflicts with the typically life-saving intent implicit in the critical care environment. The review to follow dissects the conflict and presents novel and alternative therapies for the purposes of improving oxygen delivery.

## 2. Anaemia in Critical Care

Anaemia is a common condition in the ICU. Data indicate that 35%–50% of all patients admitted to ICUs receive on average 5 RBC units during their ICU stay [2]. As well as its ubiquitous presence in the critically ill patient, anaemia is often complicated by a multi-etiological nature, whether this is acute haemorrhage, phlebotomy, or failure to produce erythropoietin.

Definitions of anaemia from the World Health Organisation (WHO) are a haemoglobin (Hb) level less than 13 g/dL (grams per decilitre) in men and less than 12 g/dL in women [3]. More than 90% of critically ill patients in intensive care units are found to have a below normal haemoglobin level by day three of their ICU stay [4].

The definition of life-threatening anaemia has not been fully elucidated. Multiple landmark trials recommend the use of restrictive transfusion thresholds in critically ill patients, although in JW patients it is inapt to comment on transfusion thresholds. It is well understood that a trespass of these thresholds often results in great physiologic compromise [5–8].

Treating patients with transfusion objections can be clinically demanding. The financial implications of treating patients with transfusion objections have not been thoroughly elucidated in literature. Allogenic blood transfusion entails a costly process of acquisition, storage, delivery, and complication management. Although the cost of blood conservation techniques is burdensome, the inclusive cost of allogenic blood transfusion is not insignificant [9, 10]. ICU strategies to prevent anaemia may include haematinic supplementation; local treatment of occult blood loss; and limitation of phlebotomy. These and other strategies may be successful in preventing mild or severe anaemia if applied consistently.

In contrast, life-threatening anaemia does not have such an effective armamentarium. Patients with active haemorrhage and/or inadequate tissue perfusion due to lack of oxygen delivery would be considered life-threatening anaemia. Life-threatening anaemia is best treated with allogeneic transfusions of red blood cells, which is not without risk [11]. This review explores the alternatives to this mainstay therapy, with few of these options available to the critically ill JW patient.

## 3. Physiological Changes in Response to Anaemia

In healthy individuals without anaemia, the ratio of oxygen delivery to oxygen consumption (DO_2_ : VO_2_) is more than 5 : 1 [12]. The goal in critically ill patients is to maintain a ratio of 5 : 1, or at least above 2 : 1, the point at which oxygen demand (VO_2_) becomes dependent on the supply [13]. Any reduction in the oxygen carrying capacity of circulating blood results in compensatory physiological changes. This increase in oxygen delivery occurs primarily through mechanisms to increase cardiac output (CO) [12].

The net effect of the increased cardiac output culminates in decreased blood viscosity, increased stroke volume, and vasodilation, which act together to increase red cell blood flow velocity [14, 15]. This reduction in the concentration of deoxygenated Hb further perpetuates the tissue oxygen deficit.

The body has other systemic responses to anaemic hypoxia to improve oxygen delivery. An increase in 2,3-diphosphoglycerate (2,3-DPG) production via glycolytic pathways stabilises deoxyhaemoglobin in a tense state, resulting in decreased affinity for oxygen and an increase in release of oxygen to tissues [16]. Nitric oxide-mediated regional vasodilatation in highly metabolically active tissues results in improved end organ flow. This response becomes systemic in anaemic patients with resultant high cardiac output state due to decreased vascular tone and viscosity [17, 18].

Additionally, when the abovementioned compensatory physiological changes fail to maintain the DO_2_ : VO_2_ ratio above 5 : 1, anaerobic metabolism increases with resultant accumulation of lactate and hydrogen ions [12, 19, 20]. A combination of increased lactate and worsening acidosis leads to reduced myocardial function and decrease in compensatory cardiac output, leading to lower DO_2_ [12, 21].

The critical point at which oxygen availability for aerobic metabolism becomes supply dependent is a DO_2_ : VO_2_ ratio of 2 : 1 and is estimated to reflect a Hb of 4 g/dl [22]. Experimental data have shown that until the ratio decreases beyond this point, VO_2_ remains relatively unchanged [22].

The critically ill anaemic patient is often found to have other physiological derangements. These patients have an increased resting tissue oxygen demand (VO_2_) as a result of systemic inflammation, derangements in acid/base status, and/or increases in endogenous or exogenously administered catecholamines [12]. Due to this increased VO_2_, the compensatory increase in cardiac output in order to maintain a DO_2_ : VO_2_ ratio of ≥5 : 1 will need to be of a greater degree than in non-critically ill individuals [12]. This compensation is often impaired due to a myocardial dysfunction related to critical illness [23].

The most efficient treatment to attenuate these compensatory responses and to restore adequate physiological responses is red blood cell transfusion [24].

## 4. Treatment Options

It is imperative for clinicians to establish JW patient preferences regarding RBC transfusion and all other treatment strategies to optimise outcomes early in the clinical course. Individuals within the JW community may accept certain treatments, such as intraoperative blood salvage with immediate transfusion, whereas others may not. For many, the critical factor in accepting autotransfusion is whether the system is “closed,” with continuity of the circuit and patient at all times. By ensuring this principle of keeping the blood in continuity with the patient, acute normovolemic haemodilution is often acceptable to a Jehovah's Witness.

This issue is particularly relevant in emergent and elective surgical procedures associated with large blood loss; however, these options are rarely possible in times of acute unanticipated haemorrhage.

Haemostasis is imperative to ensure the successful treatment of acute bleeding and anaemia in patients who refuse blood product transfusion. Once this has been achieved, the key management principle is the maximisation of oxygen delivery via alternative methods, allowing time for recovery of intrinsic haemoglobin production [25]. This armamentarium will be explored in three sections: optimisation of the body's oxygen carrying capacity; cardiac output; and erythropoietic stimulating agents.

### 4.1. Physiological Optimisation

#### 4.1.1. Oxygen

One of the essential goals of treatment in critically ill patients is to re-establish the delivery of oxygen (DO_2_) to the tissue [26]. The oxygen carrying capacity of blood consists of both free and bound haemoglobin, with 98% of oxygen bound to haemoglobin [27]. Due to the small proportion of dissolved oxygen, manipulation of the inspiratory fraction of oxygen or atmospheric pressure remains insignificant [27]. Despite this fact, increasing the dissolved fraction maximally should be considered a priority. The global DO_2_ would thus be determined by cardiac output, saturation, and haemoglobin [19].

Despite the above, hyperbaric oxygen therapy remains a treatment option for Jehovah's Witnesses with profound hypoxia and successful use has been described in severely anaemic patients. This treatment modality lends itself to a number of difficulties [29].

The case above reports success with the use of dissolved oxygen therapy but does not lend itself to the optimisation of haemoglobin. Physiological optimisation would generally include manipulation of haemoglobin, in an attempt to improve oxygen carrying capacity. Hyperbaric oxygen therapy should be considered in life-threatening scenarios in the JW patient, where other treatment modalities have failed, given its difficulties of implementation and other available options.

The benefits of oxygen supplementation are limited; consequently, greater scientific strides are observed in the manipulation of cardiac output in order to improve DO_2_ [19].

#### 4.1.2. Optimising Cardiac Output

Optimisation of cardiac output (CO) is not a simple task. Increases as well as decreases in CO happen rapidly in critically ill patients and overzealous augmentation of CO to above normal levels may lead to an increase in mortality in the critically ill [30].

### 4.2. Fluid Therapy and Inotropes

Most guidelines promote the judicious use of both balanced crystalloid and synthetic colloid solutions to replace lost blood volume. The interplay between preload, afterload, and contractility influences the stroke volume with the product of the stroke and HR relating to the cardiac output. Appropriate fluid therapy ensures adequate preload to the heart which is necessary to optimise cardiac output. The patient's position on the Frank–Starling curve would determine the patient's responsiveness to fluids. Alone, these fluids may not be adequate to replace the lost volume due to their rapid redistribution into the intravascular space [26]. Additionally, no crystalloid or synthetic colloid is an equivalent replacement for blood.

The mainstay of fluid resuscitation is a goal-directed therapy which should be initiated prior to the development of organ failure [27]. The concern with non-restrictive fluid therapy in these situations is that it may cause further haemodilution in an already anaemic patient, and once the haemoglobin drops below 5 g/dl, this results in a decrease in tissue oxygenation. Impaired tissue oxygenation has severe sequelae which include impaired wound healing, organ dysfunction, and coagulation abnormalities and is associated with a significant increase in mortality [25, 31]. Early addition of inotropic agents is advised and may decrease the amount of fluid administered, minimizing the total hemodilutional effect.

### 4.3. Optimising Oxygen Demand

The primary goal is to maintain or decrease oxygen demand (VO_2_), and thus when there is a decrease in DO_2_, there should not be an increase in oxygen extraction in order to cope with those demands [19, 26]. Many factors increase VO_2_ and these factors can be manipulated with mechanisms including shivering, hyperthermia, increased sympathetic drive secondary to anxiety and pain, nutrition containing a high glucose load, and exogenous catecholamines [19]. Studious temperature management in the prevention of hyperthermia is critical as increase in temperature of 1-2 degrees Celsius raises the metabolic rate by up to 25% [32]. Judicious use of paracetamol and NSAIDs can assist with temperature management, although the risk of renal dysfunction with the use of NSAIDs may limit its use in the critically ill [33]. Muscle paralysis via neuromuscular blockade has been described with mechanical ventilation to reduce VO_2_ attributed to respiratory effort and shivering [34]. These therapies are not without risk. Myopathy, patient awareness, and increased thromboembolic risk deserve consideration prior to the institution of the outlined therapies [35].

Mild hypothermia has some advantages in the setting of blood product refusal, increasing the amount of oxygen dissolved in the solution and decreasing the basal metabolic rate. Reports of its efficacy in a severely anaemic patient after colectomy have been demonstrated [34]. Counterproductively, hypothermia may increase blood loss, the need for transfusion due to impaired coagulation, and increase risk of arrhythmia, and the risk versus benefit on a per-patient case basis would need to be determined before implementing such therapy, as large-scale studies have not yet determined the safety of such therapy despite its ubiquitous nature [29, 36, 37].

### 4.4. Oxygen Carrying Capacity

#### 4.4.1. Artificial Oxygen Carriers

Alternatives to blood transfusion are limited. Conceptually, the use of a synthetic or haemoglobin-based oxygen carrier is the ideal substitute for religious objectors. The two most studied options are free haemoglobin-based oxygen carriers and perfluorocarbons. Haemoglobin-based solutions are polymerised modifications of bovine or human haemoglobin added to an electrolyte solution in order increase oxygen carrying capacity of the blood. A bovine-derived product called Hemopure® is considered acceptable for use in Jehovah's Witnesses. However, it has been denied FDA approval in the USA and is only available for compassionate use under the Expanded Access program [38].

The advantages of HBOCs include non-immunogenicity, prolonged storage period of up to three years, and lack of refrigeration equipment [39]. Levy et al. demonstrated that in cardiac surgery, Hemopure® decreases the need for blood transfusion [40]. Widespread use of these agents is curtailed by reports of myocardial injury as well as the profound hypertension observed after administration.

One Hemopure® case series of four patients reported no adverse events with positive outcomes; however, these data are not supported by large randomised control trials [39, 41, 42].

Due to the potential harmful effects and the lack of robust evidence for its use, its role in the treatment of Jehovah's Witness patients would best be described as a “time-buyer.” HBOC transfusion may be considered whilst achieving adequate haemostasis or recovery of anaemia in the critical care setting.

Perfluorocarbons are an alternative to HBOCs. These compounds act as excellent solvents for dissolving gases such as oxygen. Their oxygen dissolving capacity is directly linked to the partial pressure of oxygen. Patients who receive this drug must receive high concentrations of supplemental oxygen to achieve an adequate clinical effect. Currently, there are no available perfluorocarbon compounds on the market. This is in part due to technical issues regarding storage and temperature instability. Of greater concern, adverse effects which include thrombocytopenia, fever, and an apparent increased incidence of cerebrovascular accidents limit the potential perfluorocarbon benefits [25, 42].

Novel haemoglobin-based carriers such as HemO2Life® and Hemarina® have shown promise in small animal models and are under investigation for use in organ preservation [43, 44]. Porphyrin-based oxygen carrier HemoCD® has shown promise as a totally synthetic oxygen carrier with carbon monoxide scavenging capabilities [45].

The developments in this area may add to the armamentarium used in treating life-threatening anaemia in JW patients.

#### 4.4.2. Autotransfusion Options

Blood substitutes remain a hopeful endeavour in the resuscitation of patients refusing blood products. With substitutes currently falling short, one may consider autotransfusion practices. Most of the autotransfusion options acceptable to Jehovah's Witness patients are only useful if employed before major bleeding or anaemia has occurred [46, 47]. Cell salvage is a versatile therapeutic option for haemorrhage in multiple clinical scenarios and is becoming ubiquitous in high-income countries [10].

Acute normovolaemic haemodilution (ANH) involves setting up a closed circuit in which 500–2000 ml of the patient's blood is removed into a citrate containing bag, and the deficit blood volume is replaced with crystalloid or colloid solution, resulting in a decreased haematocrit. When bleeding occurs, it will result in loss of haemoglobin diluted blood, resulting in less red blood cells lost per millilitre of blood loss. The removed blood can then be reinfused once haemostasis has been achieved, if a “closed circuit” is maintained with the patient. Although a good option, this is not accepted by all Jehovah's Witness patients as this entails a discontinuation of the patient's circulatory system. ANH has been shown to reduce the need for allogeneic blood transfusion in cardiac surgery [29, 48].

Acute hypervolemic haemodilution (AHH) is a similar technique, which achieves haemodilution via infusion of a crystalloid or colloid solution without prior withdrawal of the patients' blood. Mielke et al. compared ANH to AHH and found no significant differences in treatment effect between the two modalities [49, 50]. The risk with this technique is in patients who may not tolerate a large fluid bolus, such as those with risk for cardiac failure or pulmonary oedema [51].

These haemodilution techniques have a limited role in critical care, where most patients will not benefit from a further decrease in haematocrit.

Cell salvage, however, has a far more promising role, in that it can be continued after theatre at the patient's bedside as a part of a closed circuit between the patient's wound drain and the cell salvage machine. The use of cell salvage would be on a patient-specific basis, as the JW population has varying opinions regarding continuity of the circulatory system [47]. Safety of cell salvage devices has been improved significantly with the use of leukocyte depletion filters. These facilitate the removal of white cells, tumour cells, bacterial cells, and amniotic fluid. However, its routine use in obstetric and malignancy cases remains a risk with a less robust evidence base [52].

#### 4.4.3. Erythropoietin Stimulating Agents

Erythropoietin stimulating agents (ESAs) have been shown to be effective in increasing haemoglobin levels and reducing the need for blood transfusion in patients with chronic kidney disease and those with cancer undergoing chemotherapy [53]. Critically ill patients exhibit low erythropoietin (EPO) release with limited bone marrow response to EPO [54].

A randomised control trial in 2006 examined the efficacy of ESAs in reducing blood transfusion requirements in critically ill patients, reporting a significant decrease in exposure to blood transfusion and high haemoglobin levels compared to controls [55].

ESAs in high doses have been championed in many case studies and small observational studies claiming efficacy in in treating anaemia in Jehovah's Witness patients [31]. This is challenged by a systematic review by Heh-Foster et al. in which 76 cases of ESA use in patients who could not be transfused due to medical reasons or transfusion refusal demonstrated no association between varying doses of ESAs and improved haemoglobin recovery after 28 days [53].

In the critical care setting, one of the main concerns around ESAs is the delay in time to clinically significant effect. An increase in haemoglobin is perceived in 10 days or in other cases only after four weeks. ESAs may not have much utility in patients that have evidence of life-threatening anaemia as the time needed for a clinically significant response to anaemia to improve DO2 is not always present in critically ill patients.

An increased risk of thrombotic complications is associated with the use of these drugs, and ESAs are contraindicated in uncontrolled hypertension or any history of recent cardiac or cerebrovascular thrombosis [39]. Whether the risks of thrombosis caused by ESAs are relevant in severely anaemic patients is offset by the effects of anaemia on haemostasis [56].

### 4.5. Parenteral Iron, Folate, and B12 Replacement Therapy

It is recommended that when ESAs are given, they are combined with iron, folate, and vitamin B12 in order to maximise the erythropoietic effect. Routine folate supplementation lacks high-quality evidence in this context as further studies are needed to prove efficacy.

In the context of iron deficiency, intravenous iron therapy alone produces a significant increase in haemoglobin compared to controls. Intravenous iron therapy in the elective perioperative setting has shown benefit; however, multiple studies indicate failure of parenteral iron to reduce the risk for perioperative transfusion, especially in the emergency setting. This is considered due to the proinflammatory state that acute illness and surgery produce. The physiological stress induces the production of hepcidin, which inhibits the absorption of gastrointestinal iron, which is not applicable to parenteral iron therapy, and the release of stored ferritin into the circulation, effectively inhibiting the transport of iron to the bone marrow for erythropoiesis [57].

The concerns surrounding iron supplementation are the small risk of anaphylactic reactions and the risk of infection associated with intravenous iron therapy, although there is conflicting evidence as to whether this risk is significant or not [58–61]. In the critical care setting, where gastrointestinal absorption may be limited, intravenous iron therapy will allow a higher bioavailability of the drug. The IRONMAN trial compared anaemic patients admitted to ICU receiving intravenous iron and placebo. Despite higher haemoglobin levels at discharge, there was no difference between the groups in morbidity or mortality with no decrease in transfusion requirements [62].

As stated, ESAs should be complemented with iron, folate, and vitamin B12 in order to maximise the erythropoietic effect. Formation and maintenance of haemoglobin depend on multiple nutrient factors apart from iron and EPO. Rodriguez et al. demonstrated that up to 13% of ICU patients already have haematinic deficiency in the acute phase of admission [63].

Due to the safety profile of vitamin B12 and folate, current recommendations advocate for liberal administration to these patients; however, there is scant evidence to inform the standard of care [64].

### 4.6. Hepcidin Antagonists

Hepcidin gate keeps the release of stored iron needed for effective haematopoiesis as well as modulating the uptake of iron systemically. During inflammation, the release of iron is prohibited and erythropoiesis is curtailed by hepcidin as mentioned previously. Hepcidin antagonists are novel drugs, in multiple phases of clinical trials, that aim to interfere with the production and functioning of hepcidin via multiple pathways in order to mobilise iron stores. Complete removal of the regulatory protein is not feasible due to the consequences of iron overload and its antimicrobial effect [65]. The future role of these drugs in a critical care setting is yet to be determined [66, 67].

## 5. Conclusion and Future Perspectives

The management of critical anaemia in patients that refuse blood products remains a problem despite significant advances in technology and surgical techniques. The ideal agent to replace blood seems to be on the horizon with many options under investigation. However, in the interim, knowledge of physiological optimisation and understanding of multiple treatment modalities available to clinicians are invaluable. Artificial oxygen carriers demonstrate great theoretical value in the management of the anaemic JW patient; however, to date, these products have not proven an effective therapy in severe anaemia.

## Data Availability

This is a review article. All literature reviewed is referenced in the article.
